# Acellular dermal matrix in reconstructive surgery: Applications, benefits, and cost

**DOI:** 10.3389/frtra.2023.1133806

**Published:** 2023-03-10

**Authors:** Fatemeh Mohammadyari, Sadaf Parvin, Mohsen Khorvash, Amirhasan Amini, Amirhossein Behzadi, Reyhaneh HajEbrahimi, Fatemeh Kasaei, Sepehr Olangian-Tehrani

**Affiliations:** ^1^ School of Medicine, Guilan University of Medical Sciences, Rasht, Iran; ^2^ School of Medicine, Iran University of Medical Sciences, Tehran, Iran; ^3^ School of Medicine, Islamic Azad University of Medical Sciences, Tehran, Iran; ^4^ School of Medicine, Zahedan University of Medical Sciences, Zahedan, Iran; ^5^ School of Medicine, Tabriz University of Medical Sciences, Tabriz, Iran; ^6^ Avicennet, Tehran, Iran

**Keywords:** acellular dermal matrix, ADM, plastic surgery, wound healing, tissue engineering

## Abstract

Modern tissue engineering has made substantial advancements that have revolutionized plastic surgery. Acellular dermal matrix (ADM) is an example that has gained considerable attention recently. ADM can be made from humans, bovines, or porcine tissues. ADM acts as a scaffold that incorporates into the recipient tissue. It is gradually infiltrated by fibroblasts and vascularized. Fortunately, many techniques have been used to remove cellular and antigenic components from ADM to minimize immune system rejection. ADM is made of collagen, fibronectin, elastin, laminin, glycosaminoglycans, and hyaluronic acid. It is used in critical wounds (e.g., diabetic wounds) to protect soft tissue and accelerate wound healing. It is also used in implant-based breast reconstruction surgery to improve aesthetic outcomes and reduce capsule contracture risk. ADM has also gained attention in abdominal and chest wall defects. Some studies have shown that ADM is associated with less erosion and infection in abdominal hernias than synthetic meshes. However, its higher cost prevents it from being commonly used in hernia repair. Also, using ADM in tendon repair (e.g., Achilles tendon) has been associated with increased stability and reduced rejection rate. Despite its advantages, ADM might result in complications such as hematoma, seroma, necrosis, and infection. Moreover, ADM is expensive, making it an unsuitable option for many patients. Finally, the literature on ADM is insufficient, and more research on the results of ADM usage in surgeries is needed. This article aims to review the literature regarding the application, Benefits, and costs of ADM in reconstructive surgery.

## 1. Introduction

Traditionally, autologous tissue grafts and synthetic materials have been used in reconstruction surgery, but each has its disadvantages (1). With autologous tissue graft, the morbidity of the second (donor) surgical site and the patients' more painful recovery period were significant problems (1). While with Synthetic materials, infection is always a risk (2). Thus, reconstructive surgery still needs a suitable material (2).

Acellular dermal matrix (ADM) is a biological graft that contains neither cellular nor antigenic components to avoid immunogenicity (3). Therefore, it has been recognized as a good substitute material for plastic and reconstructive surgeries (4). ADM can be obtained from humans, bovine, and porcine tissues (2). ADM comprises collagen fibers, fibronectin, elastin, laminin, glycosaminoglycans, and hyaluronic acid (2, 5, 7, 8). It serves as a scaffold that is gradually vascularized and cellularized by the host (2, 6, 8–10). ADM is utilized in aesthetic and reconstructive surgeries of nasal and oral cavities, breast, and abdominal walls (2, 4, 10, 11). It is also used in burn and diabetic wounds (4, 6, 9).

It should be noted that ADM is not supposed to substitute a full layer of skin tissue due to its lack of epidermis (2). By using ADM, instead of a full-thickness skin graft, only a thin layer of skin graft is needed leading to less scar formation at the donor site (2).

Many studies have shown favorable results of ADM in implant-based breast reconstructions with low complication rates (2). Also, some research showed faster healing of diabetic foot ulcers with ADM than standard treatment (12). Several studies have also evaluated the outcomes of biological mesh (i.e., ADM) vs. synthetic mesh in abdominal hernia repair, but the results are contradictory, and more research is warranted (13).

Despite its advantages, ADM is still a foreign material, and infection, necrosis, and seroma are possible complications (14). Also, ADM is expensive and can only be a reasonable choice for some patients (2).

Unfortunately, the data on the clinical outcome of ADM usage in many surgeries is still insufficient (8). Research on the cost-effectiveness of ADM is also inadequate, and more work is warranted to clarify these matters (8). This review highlights ADM applications in reconstructive surgery and its costs and benefits.

## 2. Head & neck

ADM is increasingly used in cosmetic and reconstructive head and neck surgeries. It is applied in periorbital soft tissue, dura mater, extraoral and intraoral, and oropharyngeal defects. It also has applications in skeletal support, nasal soft tissue, and tympanic membrane repair (1, 15–23).

Many studies have worked on using ADM in cleft palate repair (1, 10, 17, 18, 20, 21, 23–54). Achieving a tension-free, water-tight closure has always been a challenge in palatoplasty because otherwise, the patient may predispose to a fistula formation (55–57). The use of ADM in palatoplasty has been associated with better repair and less fistula formation (55–57). In palatoplasty, a thin (nearly 0.5 mm) 2 × 4-cm piece of ADM is sutured to the nasal lining (58). These measures change due to the availability of ADMs at different surgical facilities (28, 55, 58). This technique results in a more robust repair with a lower chance of fistula formation (28). Helling et al. (58) found that in a case series of 31 ADM palatoplasties, the rate of fistula formation was lower (3.2%) compared to historical cohorts (about 8%) (58). To support these findings, additional research with bigger sample sizes is necessary (56).

The disadvantage of ADM is its high price of approximately $135. Fortunately, the significant financial burden of fistula repair surgery makes this price seem acceptable (55, 58). Aside from the cost, ADM is an avascular graft, which means that necrosis and infection are its potential consequences (28).

Skull base tumor excision has always been challenging (1, 10, 17, 18, 20, 21, 23–54). Even with the endoscopic excision, the risk of complications (e.g., rhinorrhea) remains high (59, 60). As a result, identifying an appropriate material for CSF rhinorrhea repair following tumor excision is critical (10, 18–20, 22–24, 26–28, 30–54, 61–70). In a randomized control trial study, Zhong et al. (59) compared the clinical outcome of CSF leakage repair using ADM with Turbinate Flap. They used a fascia lata graft to repair the defect in the dura mater. Then they covered the fascia lata with ADM and adhered the ADM to the nasal mucosa. Eventually, Vaseline gauze is inserted into the nasal cavity (59). They found that both ADM and the turbinate flap had corresponding clinical outcomes. Thus, ADM might be a safe alternative for CSF rhinorrhea repair following skull base tumor excision (59).

Youngerman et al. (71) also compared the ADM and autologous fascia lata graft in CSF leakage repair. They opted to use ADM or fascia lata to repair the dural lesions with substantial CSF leakage. The ADM or fascia lata was then covered with a polyethylene implant.

They discovered similar clinical outcomes in the two groups. Accordingly, ADM is a viable option for this operation since it eliminates the requirement to obtain an autologous fascia lata graft from the patient's tissue (71). Also, Mericli et al. (72), in a case report, used a 4 × 1 cm piece of ADM with a thickness of 1–2.3 mm for CSF repair and found a successful result (72). These measures depend on the patient's needs and the surgical approach (72).

To confirm these findings, more research on the efficacy and cost-effectiveness of ADM in CSF rhinorrhea repair is needed (71).

Nasal septal perforation management remains a serious issue in otorhinolaryngology (18, 23, 25, 28, 30, 31, 33, 36, 38–54, 60, 64, 68–70, 73). Several treatment strategies have been presented so far, but their success rate is debatable (25). Prosthetic nasal buttons are a non-invasive form of treatment, although they are often poorly tolerated by patients (25). Conrad et al. (25) employed an ADM graft as a substitute material for septal perforations (25). They demonstrated that ADM administration is an effective approach for minimizing postoperative symptoms (nasal obstruction, sleep issues) (25). Nevertheless, there is insufficient data to support this finding (25, 41).

The use of ADM in the treatment of rhinophyma has recently attracted interest (74, 75). Torresetti et al. (74), in a case report, described the use of an ADM with the size of 5  ×  5-cm on a severe rhinophyma. Postoperatively, the nasal shape and functions had improved, but the aesthetic results were unsatisfactory, and the patient still needed cosmetic procedures (74). They suggest that despite ADM's efficacy in rhinophyma surgery, more aesthetic procedures may be needed (74). Another factor that physicians should consider is the higher expense of ADM compared to other surgical procedures (74).

On the contrary, Ozkan et al. (75) used ADM in another severe rhinophyma case and found a successful aesthetic and functional result (75). As the results are inconclusive, additional research is required to shed light on this topic (75).

Recent studies have shown favorable results regarding the use of ADM in Tympanic membrane reconstruction (10, 18–20, 22–24, 26–28, 30–54, 61–70).

**Figure 1:**
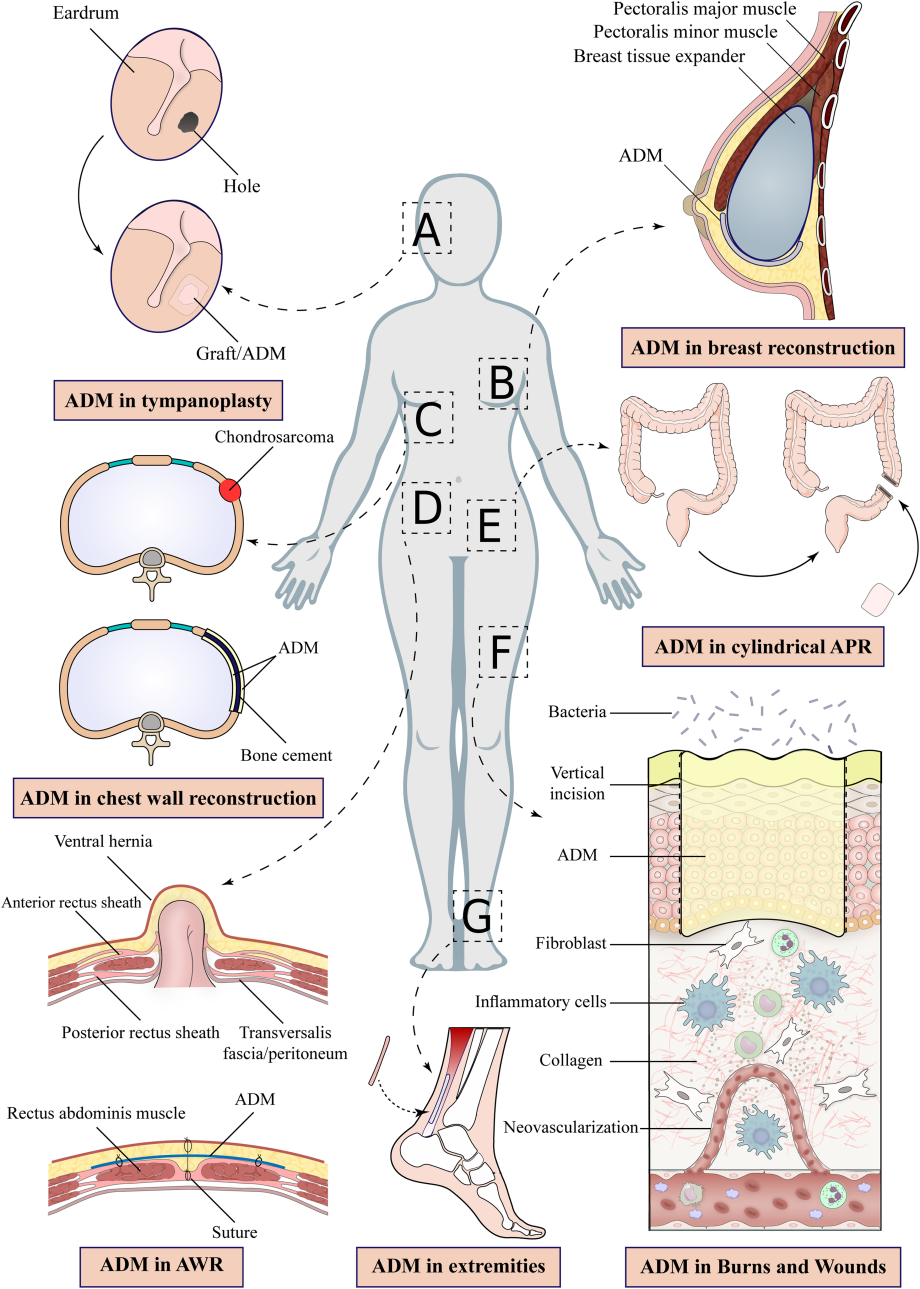
ADM Application in human body reconstruction.

Lee et al. (76), in a clinical trial, compared the results of tympanoplasty using ADM to the standard approach (using tragal perichondrium) (Figure 1A) (76). For the ADM group, they used an ADM graft with a size of 1.5 × 2-cm and a thickness of 0.3–0.5 mm (76). They demonstrated that ADM yielded similar hearing outcomes and a shorter surgical duration than conventional methods (76).

Research has suggested that ADM is a suitable material for rhinoplasty (18, 22, 23, 25, 28, 30, 31, 33, 36, 38–54, 60, 62, 64, 68–70, 73). Previous studies have shown a low rate of extrusion and infection in ADM-used rhinoplasties (77). Park et al. (78) have studied the use of ADM in primary and revision dorsal augmentation rhinoplasties. They showed that ADM can be used in both situations and has a low complication rate (78). Other studies have shown that ADM is a biocompatible material that gives a natural appearance to the nose, long-term integrity, and low infection and extrusion risk (77).

In a retrospective study, Yang et al. (79) assessed the utility of ADM in dorsal augmentation rhinoplasties (79). They stated that ADM is an ideal substitute for autologous tissue due to the reduction of donor site morbidity. They showed successful results of ADM with the patient satisfaction score of 81.02 out of 100. Moreover, none of the patients experienced any major complications (79).

In a case series, Sherries et al. (80) investigated the complications of ADM in rhinoplasty. They found no infection, skin discoloration, seroma, septal perforation, and extrusion (80). According to their study, ADM is a safe and effective substitute for traditional methods (80).

## 3. Chest

Chest wall defects are frequently caused by tumor herniation and resection (1, 2, 21, 29, 30, 81–91). Defects in the chest wall impair the strong framework that supports breathing and safeguards the viscera (92). Loss of chest wall integrity leads to devastating complications such as lung hernia, hemithorax shrinkage, and paradoxical chest wall motion (50). Consequently, it is essential to repair the chest wall properly (50). The material typically employed in chest wall repair is synthetic mesh (1, 2, 21, 29, 30, 81–91). It does, however, have drawbacks (for example, infection) (93). ADM has so been the subject of investigations to replace the mesh in these operations (Figure 1D) (1, 2, 21, 29, 30, 33, 36, 68, 81–91, 94–101).

In a study, Heo et al. (29) did chest wall repair with ADM on six patients following tumor resection. This technique employed two layers of ADM and bone cement (29). They discovered positive outcomes, and none of the patients experienced postoperative complications or soft tissue defects (29). They demonstrated the safety of ADM and its substitutability in chest wall repair after tumor removal (Supplementary Table S1) (29).

Also, Giordano et al. (93), in a study, compared the postoperative complications of mesh vs. ADM in chest wall reconstruction. They discovered a lower rate of surgical site infection in the ADM group, demonstrating ADM's suitability for patients at higher risk of infection (93).

Yoon et al. (102) also used ADM to repair a chest wall defect caused by a sternal metastatic tumor excision (102). To repair the defect, a 12 cm × 12 cm piece of ADM was used in this technique. The patient had a successful clinical outcome, and no complications emerged (102).

Rigid prosthetics were previously used for large chest wall defects (89). These prosthetics were inflexible, resulting in reduced lung capacity and even pain. As foreign bodies, they also increased the likelihood of infection (89). Following chondrosarcoma resection, Ely et al. (89) decided to repair a sternal defect with an ADM and soft tissue flap. The patient had excellent clinical outcomes with no complications over a two-year follow-up (89). They showed that ADM as a biological mesh could repair large sternal defects (Figure 1C) (89).

Stanizzi et al. (103) used ADM in a lung hernia and severe pectoralis major muscle retraction following a mini-thoracotomy for mitral valve surgery (103). The lung hernia was reduced following surgery, and it did not recur during a six-year follow-up (103). They discovered excessive serum production after surgery, so the patient had a drain for 15 days to avoid seroma. As a disadvantage, ADM is more expensive than synthetic meshes or autologous tissues, which keeps it from being a popular repair method (103).

## 4. Breast

Breast cancer is the most common cancer in women worldwide, causing numerous social and psychological issues (104–106). Several surgical options are being employed for breast reconstruction surgery. The use of ADM in implant-based breast reconstruction following mastectomy has received a lot of attention recently (2, 83, 91, 94, 104, 107–112). The advantages of ADM in implant-based breast surgery include implant stabilization, improved aesthetic outcomes, and reduced capsular contracture risks, donor site morbidity, and postoperative pain (34, 83, 86, 109, 112–114).

Following a mastectomy, implant-based breast reconstruction is a popular option (Figure 1B) (83). Yet, one of the risks of this technique is capsular contracture (115). Previous research has suggested that ADM-covered breast implants are less prone to capsular contracture (115). Stump et al. (115) decided to compare the capsular contracture rate in breast implants with and without ADM in primates (115). They found that ADM-covered breast implants significantly reduce the rate of capsule contracture. Enclosing the implant in ADM prevents the immune system from recognizing it and forming a fibrous capsule around it (115). More research is needed to determine whether this result is repeatable in humans (115).

As previously stated, ADM is a costly option compared to other reconstructive materials (116). In a cost analysis study, Jensen et al. (116) compared the cost of implant-based breast reconstruction with and without ADM. Surprisingly, they discovered that implant-based breast reconstruction with ADM is less expensive ($10,240 vs. $10,584 for a 6 cm × 16 cm ADM sheet). With a more miniature ADM sheet (6 cm × 12 cm), the cost drops to $9673 (116). These estimates were derived from a university-based hospital in Canada, and more research on different medical centers is needed to prove this cost-effectiveness (116).

Nevertheless, the use of ADM in implant-based breast reconstruction has disadvantages (114, 117).

The most frequently mentioned ADM complications in the literature are necrosis, seroma, hematomas, and infection (2, 104, 118). In a randomized control trial, Hansson et al. (119) compared the first-year complication rate of a biological mesh (ADM) with that of a synthetic mesh in implant-based breast surgeries (119). They found a higher implant loss rate due to infection in ADM cases than in synthetic mesh cases (12.5% vs. 0%). They hypothesized that this increased infection rate was due to higher seroma formation in ADM patients (38% vs. 3.8%) (119).

Also, Dikmans et al. (120), in a randomized control trial, investigated the rate of adverse events of implant-based breast reconstructions with and without ADM. They showed that skin necrosis (11% vs. 1%), Hematoma formation (3% vs. 2%), and wound infection (8% vs. 2%) were all higher in the ADM group. According to their findings, the ADM group had a lower rate of seroma formation (3% vs. 2%), which contradicts previous research (120).

Lohmander et al. (99) also compared the complications of implant-based breast surgeries with and without ADM. They discovered that the ADM group has a higher rate of skin blisters (9% vs. 0%) and infection rates (14% vs. 6%). In another study, Kumar et al. (96) also suggested that ADM is associated with a higher rate of postoperative complications, especially in overweight women (96).

Some patients develop erythema following ADM breast reconstruction surgery (118). This condition is known as red breast syndrome (118). Danino et al. (118) investigated red breast syndrome cases and found bacterial biofilms on all the ADMs, hypothesizing that these biofilms could be linked to the syndrome (118).

## 5. Abdomen

Despite numerous options for abdominal wall reconstruction, surgeons have long struggled to find an ideal mesh (1, 16, 21, 29, 83, 87, 89, 121–126).

In the past, abdominal hernias were treated with synthetic meshes such as polypropylene (82). Despite their strength, their complications, like erosion into the bowel wall, adhesion, infection, and fistula formation, have limited their use in abdominal wall reconstruction (81). So, biological meshes such as ADM have recently gained attention (1, 16, 21, 29, 33, 34, 38, 45, 83, 87, 89, 101, 121–126, 127). ADM is biocompatible, integrates into the surrounding tissue, and allows vascularization (1, 16, 21, 29, 33, 34, 38, 45, 83, 87, 89, 101, 121–126, 127). The revascularization of ADM by the host makes it resistant to infection (1, 10, 16, 21, 29, 33, 34, 38, 45, 52, 83, 87, 89, 101, 121–126, 127–129).

Cevasco et al. (130) reviewed the pros, cons, and indications of using different meshes (130). They showed that propylene meshes are long-lasting and suitable for use in extra-peritoneal clean hernias (130). Due to their high rate of erosion into the bowel wall, they are not recommended for intraperitoneal placement (130). Still, their low cost and easy application make them a good choice for many surgeries.

Cevasco et al. (130) also showed that ADM is a suitable substitute in contamination where other synthetic meshes are not recommended (130). Unfortunately, ADM costs more than synthetic mesh, preventing its widespread use (131).

In a systematic review, Fischer et al. (132) investigated the cost-effectiveness of different meshes in clean-contaminated hernias (132). Their study showed that synthetic mesh costs $15,776, while biological mesh costs $23,844 (132). In clean-contaminated hernia repair, synthetic mesh is still a cost-effective material (132). This view has been corroborated by other studies (133).

The data on the recurrence rate of hernia repair using ADM is scarce. Brewer et al. (134) showed that biologic mesh (ADM) has a lower recurrence rate than non-biologic mesh (24% vs. 77%) (134). In contrast, Darehzereshki et al. (135) found no significant difference between biologic and non-biologic mesh recurrence rates (135).

Garvey et al. (136) found that using ADM for abdominal wall reconstruction resulted in 11.5% and 14.6% hernia recurrence rates after 3 and 5 years of follow-up, respectively (136).

More research is needed to determine the precise contribution of ADM to abdominal wall reconstruction (137).

## 6. Pelvis

There are various surgical methods for treating urogynaecological disorders, each with its own set of advantages and disadvantages (138). Synthetic materials can cause erosion, infection, hematoma, and dyspareunia (34, 46, 60, 121, 123, 129, 139–141). ADM is a biocompatible material that may be linked to a lower risk of complications, increased durability, and improved efficacy (34, 46, 60, 121, 123, 129, 138–141). The host vascularizes ADM sheets, lowering the risk of infection and making it an ideal choice for infected surgical sites (142).

Many studies have confirmed the benefits of ADM in vaginal reconstruction surgery (34, 46, 53, 54, 60, 121, 123, 129, 139–141, 143, 144). Gualtieri et al. (145) compared the effect of propylene mesh with porcine ADM on vaginal smooth muscle cells. They showed that vaginal smooth muscle cell proliferation is higher on ADM compared to synthetic mesh. This could explain why ADM produces better results and has a lower erosion rate than synthetic mesh (145).

ADM has also been used in the treatment of abdominoperineal resection, urogynaecological issues, and perineal wound complications (146, 147).

In a study, Han et al. (146) investigated the surgical complications in pelvic reconstruction using ADM following cylindrical abdominoperineal resection (Figure 1E) (146). In 11 patients, the perineal wounds healed two weeks after the operation. After a median follow-up of eight months, they concluded that ADM is a safe and suitable alternative for reconstructing large pelvic defects in patients after cylindrical abdominoperineal resection (146).

Tognetti et al. (147) concluded that synthetic materials are not recommended after some cases of pelvic exenteration and radical vulvectomy and that ADM is a suitable alternative in these cases (147).

ADM can have some negative side effects, such as hernia bulging, infection, seroma, and chronic pain (148–150). Various studies have found that the rate of seroma production ranges from 6% to 26% (146) (146). Unfortunately, there is insufficient data on the complications of ADM in pelvic reconstruction (151). According to Han et al. (146), despite the successful use of ADM in pelvic reconstruction, nearly 33% of patients experienced chronic pain and discomfort (146). This pain could also be attributed to the surgical procedure and wide excision (146, 151). The presence of chronic pain following biologic mesh has been echoed by other studies (151, 152).

Butler et al. (153) studied the use of ADM in pelvic reconstruction in cancer patients. They used an average of 4.5 (2 to 10) ADM sheets per defect (defect size approximately 435 cm^2^). They discovered that ADM had positive results for pelvic reconstruction, but its high cost prevents it from being used in many cases (153). Another potential disadvantage of ADM is its limited size. The largest available ADM is 8 × 12-cm, and for larger defects, ADM sheets need to be joined together (153). More research is required to elucidate the benefits of employing ADM over synthetic mesh (153).

ADM can manage complex pelvic defects by forming a barrier between the intra-abdominal contents and external flaps, preventing intestinal adhesion, obstruction, and fistula (50). There need to be more extensive trials comparing synthetic mesh to biological mesh and more studies on the cost-analysis of the ADM (151).

## 7. Extremities

In recent years, the use of ADM in extremity reconstructive surgery has increased (1, 10, 16, 19, 26, 30, 40, 46, 47, 62, 73, 97, 124, 139, 140, 154–158). ADM's applications in the extremities include soft tissue and tendon regeneration, heel and nail bed reconstruction, and burn and diabetic wound management (Figure 1G) (50).

Despite its thickness, the Achilles tendon can be torn and damaged (159, 160). To repair an Achilles tendon rupture, various augmentations such as autografts, xenografts, and allografts are used. Autografts are associated with complexity, donor site morbidity, and longer surgery time. Xenografts are associated with rejection risk. These issues have prompted some surgeons to abandon their use in favor of alternative materials such as ADM (159, 160).

Tendon repair has been successful with the use of ADM (1, 10, 16, 19, 26, 30, 40, 46, 47, 52, 54, 62, 73, 97, 124, 139, 140, 154–158, 161–163). ADM is an ideal scaffold for native cells and is ready to be vascularized by the host (40, 159). In a case series, Cole et al. (160) used a 5 cm × 5 cm piece of ADM for tendon augmentation and found successful results, with no complications or rerupture (160).

In the upper limb, lee et al. (163) conducted a randomized trial on the use of ADM in the repair of the flexor tendons of the III, IV, and V sections of the hand (163). They applied an ADM sheet over the repaired tendon and found good functional results with no peritendinous adhesions postoperatively (163). This study shows that ADM might act as an anti-adhesive physical barrier in hand tendon repair. They calculated that using ADM increases the cost of surgery by 15%, but since it cuts down on the need for reoperation (due to postoperative adhesions), this may be a fair trade-off (163). More studies with larger sample sizes are needed to corroborate this data (163).

In lower limb reconstruction, heel reconstruction is one of the most challenging procedures. Many studies have found ADM to be effective in heel reconstruction (1, 10, 16, 19, 20, 26, 30, 40, 46, 47, 52, 54, 62, 73, 97, 124, 139, 140, 154–158, 161). A case report of heel reconstruction using ADM and skin graft showed optimal gait function recovery and social participation (157). According to gait analysis, measured gait and posture were essentially normal with ADM (157). Nonetheless, the pressure distribution study revealed a slight imbalance, which could be attributed to the new morphology and sensitivity of the feet (157).

Fingertip injuries are common and can result in nail trauma; however, nail bed repair is challenging due to the exposed bone (164). ADM and subsequent skin graft can be utilized to repair nail bed injuries and loss of germinal matrix. Unfortunately, patients with complex crush trauma may not be good candidates for this method (164). In a study, Fiedler et al. (165) successfully used a monolayer bovine ADM to reconstruct a sterile nail bed (165). Still, they did not recommend this technique for nail beds with germinal matrix injuries (165). The cost of the specific ADM used in this study (single layer, 5.08 cm × 5.08 cm) is $2,266, making it impractical to use widely (165).

Management of extremity burn wounds is critical due to the risk of systemic infections and death (166–168). ADM can be utilized effectively in extremity burn repairs by minimizing wound contractions, lowering the risk of recurrence, and improving function (166–168). Additionally, it can be underlined that ADM grafts can be used on nerves, arteries, and tendons where skin grafts cannot integrate and result in contractures (167). It should be noted that ADM is not recommended in cases of compromised tissue vasculature or infection (167). In addition, Diabetic foot ulcers must be managed due to the risk of infection, amputation, and death (12, 169–172). Numerous articles have discussed the use of ADM in these types of wounds, and it has been shown to be effective in closing Diabetic foot ulcers (12, 169–172).

## 8. Burns and wounds

Severe burns can cause serious skin damage, and managing the aesthetic outcomes and potential infections of these injuries is critical (16, 21, 24, 31, 33, 44, 62, 64, 66–68, 99, 123, 139, 140, 147, 154, 162, 173–176). Full-thickness and some deep partial-thickness burns require skin transplantation (177–180). Split-thickness skin grafts, local flap coverage, and skin substitutes are materials used in transplantation. The disadvantages of these materials include morbidity of the donor site, risk of flap and graft complication, and failure.

As an acellular skin substitute, ADM incorporates into the wound and is gradually vascularized by the wound bed allowing infiltration of fibroblasts (16, 21, 24, 31, 33, 44, 62, 64, 66–68, 99, 123, 139, 140, 147, 154, 162, 173–176). These events protect the wound and prevent granulation tissue and scar formation (Figure 1F) (181).

Recently, self-assembled skin substitutes have been used to manage severe burn wounds. A Self-assembled skin substitute is a dermal substitute underlying an epidermis (182). Producing these materials is troublesome and takes nearly four weeks (182). According to Cloutier et al. (182), the addition of ADM to self-assembled skin substitutes reduces production time, increases cell proliferation, and reduces the possibility of rejection (182).

In a randomized control trial, Heimbach et al. (183) compared the result of ADM graft vs. conventional skin graft material (183). In the intervention group, they sutured the artificial dermis (ADM) to the wound, and 14 days later, when it was revascularized, they grafted it with a thin epidermis. They showed that the functional and aesthetic results were comparable (183). They found that the ADM group had a less hypertrophic scar, and the patients were more satisfied with the ADM (183). When compared to other methods, ADM requires a thinner layer of the epidermis, resulting in faster donor site healing (183).

Moreover, Demircan et al. (184) conducted a study on multiple burn wounds to confirm the positive outcomes of ADM and showed a successful graft outcome (184). They stated that the graft was comparable to normal skin regarding vascularization, elasticity, plasticity, texture, and color (184). They also came to promising results in terms of aesthetic and practical dimensions. This result confirms the influential role of ADM in dermal replacement (184). ADM is associated with rapid healing and satisfactory aesthetic and functional outcomes (184, 185). There is also the possibility of favorable outcomes following the closure of facial burn wounds in children (184).

Other studies on the effects of ADM on deep burns have yielded promising results (16, 21, 24, 31, 33, 44, 62, 64, 66–68, 99, 123, 139, 140, 147, 154, 162, 173–176).

Zhi-Qian Guo et al. (186) reported a combination of early dermabrasion and porcine ADM coverage could facilitate the healing of the wounds. It also reduces hospitalization time and improves the aesthetic and functional outcomes of extensive deep dermal burns (186).

Yet, there needs to be more information concerning the contribution of ADM in burn wound management (187). Given the high price of ADM sheet, more study on the cost-effectiveness of ADM in burn wounds needs to be done (187).

## 9. Conclusion and future perspective

Using ADM in various surgical procedures can yield favorable results in function, aesthetics, and fewer complications. ADM is an acellular graft that protects it against immunogenicity. It also spares the need for extracting the autologous graft, reducing the morbidity of donor-site surgery. ADM is rapidly vascularized and cellularized by the host. This mechanism protects it against infection and makes it ideal for contaminated sites.

Previous literature results have shown successful results of ADM use in palatoplasty, implant-based breast surgery, tendon repair, and wound management. However, the results of ADM use in rhinophyma and hernia repair have been contradictory.

Despite all the ADM benefits, complications such as hematoma, seroma, necrosis, and infection must be considered. Moreover, ADM is an expensive material and cannot be used ubiquitously. More work should be done to achieve cheaper ADM to make it a cost-effective choice. Overall, the number of reports on ADM is limited, and more extensive research on ADM use, especially in plastic and reconstructive surgery is expected.
