# Supplementary material for: Acellular dermal matrix in reconstructive surgery: Applications, benefits, and cost
Source: Front Transplant. 2023 Mar 10;2:1133806. doi: 10.3389/frtra.2023.1133806 (PMC11235262; doi:10.3389/frtra.2023.1133806)
Supplement: Supplementary file 1 [file Table1.docx]

| **Authors, Year** | **Product** | **Origin** | **Surgical Site** | **Number of patients** | **Outcome** |
| --- | --- | --- | --- | --- | --- |
| Helling et al ., 2006 (1) | AlloDerm | Human | Palate | 31 patients | A lower fistula formation rate was seen compared to traditional methods. |
| Mirzai et al., 2021 (2) | AlloDerm | Human | Nasal Septal | 5 patients | No crusting, empty nose syndrome, postoperative hemorrhage, further procedures, recurrence or persistent perforations, or other problems.  All patients’ preoperative symptoms had improved. |
| Zhong et al., 2019 (3) | Heal-All (Yantai Zhenghai  Bio‐technology Ltd Co.) | Animal (bovine) | Nasal cavities | 31 patients | No evident side effects or problems.  At eight weeks, Lund-Kennedy scores in the ADM group were considerably lower than those in the control group.  The ADM group’s eight-week epithelialization period was substantially shorter than the 14-week control group. |
| Conrad et al., 2018 (4) | AlloDerm | Human | Nasal Septal | 12 patients | Complete closure of the septal hole was effective in 10 of 12 patients. |
| Zhong et al., 2019 (5) | AlloDerm | Human | Skull base | 46 patients | ADM's postoperative results were similar to Turbinate Flap's.  ADM may be a safe and practical option for endoscopic CSF rhinorrhea correction following skull base tumor resection.  In both groups, no recurrence was identified. |
| Youngerman et al., 2020 (6) | AlloMAX | Human | Skull base | 19 patients | ADM is as effective as fascia lata graft in rhinorrhea repair. It is also associated with a reduced risk of donor site morbidity compared to the traditional method. |
| Lee et al., 2018(7) | MegaDerm | Human | Tympanic membrane | 60 patients | ADM was associated with successful results. both group (ADM versus tagal perichondrium) had comparable results regarding graft success and hearing. Moreover, ADM was associated with a shorter surgery time |
| Park et al., 2022 (8) | AlloDerm | Human | Nasal cavity | 145 patients | The study showed that ADM is an ideal substitute for primary and revision dorsal augmentation rhinoplasty. Also, ADM was associated with low complications. |
| Yang et al., 2018 (9) | MegaDerm | Human | Nasal cavity | 18 patients | They showed that ADM is a suitable substitute for autologous materials. It has a similar success rate with a very low complication rate. It also eliminates the donor site morbidity associated with autologous grafts. |
| Heo et al., 2022(10) | AlloDerm | Human | Chest wall | 6 patients | ADM cannot offer sufficient stiffness to preserve thoracic organs. Hence bone cement is applied between ADM.  Primary closure was accomplished on exterior wounds in all patients without overlaying soft tissue defects.  There were no significant complications found. |
| Giordano et al., 2020 (11) | STRATTICE | Animal tissue (porcine) | Chest wall | 146 patients | The patients in the ADM group had less surgical site infection rate than the synthetic mesh group. |
| Hansson et al., 2021 (12) | Veritas Collagen Matrix | Animal tissue (bovine) | Breast | 24 patients | ADM was associated with a higher implant loss infection rate than synthetic mesh. |
| Dikmans et al., 2017 (13) | STRATTICE | Animal tissue (porcine) | Breast | 142 patients | ADM was associated with more complication rates, including higher skin necrosis, hematoma, and wound infection. However, the seroma rate was lower in the ADM group. |
| Tierney et al., 2022 (14) | SimpliDerm - a novel human ADM and AlloDerm Ready-To-Use (RTU) - an established ADM | Human | Breast | 107 patients | 27 patients (25.2%)  suffered from total of 35 side effects including infection(22.9%), flap ischemia(25.7%) and seroma(14.3%) |
| Negenborn et al., 2019 (15) | STRATTICE Reconstructive Tissue Matrix (RTM) | Animal  Tissue  (porcine) | Breast | 59 patients | Surgical complications in one-stage implant-based breast surgeries with ADM group were about 3 times more than in the two-stage implant-based breast surgeries group.  The usage of ADM was accompanied with improved aesthetic outcomes. |
| Jones et al., 2019 (16) | AlloDerm  RTM | Human | Breast | 94  patients | Better clinical and functional outcomes, minimum pain, and improved convenience in patients using ADM |
| Brunbjerg et al., 2021 (17) | STRATTICE | Animal  Tissue  (porcine) | Breast | 44 patients | High levels of satisfaction with aesthetic outcomes in patients using ADM  Attain an implant-based breast reconstruction with lesser surgeries and outpatient visits in patients using ADM. |
| Khan et al., 2021 (18) | Surgimend | Animal tissue  (bovine) | Breast | 65 patients | Low complications and reconstructive failure rates were associated with pre-pectoral implant-based breast surgeries utilizing ADM in patients. |
| Lohmander et al., 2019 (19) | STRATTICE  Reconstructive Tissue Matrix (RTM) | Animal  Tissue  (porcine) | Breast | 64  patients | implant-based breast surgeries with ADM group experienced more overall complications and reoperations and higher risks of wound healing complications in comparison with the group undergoing implant-based breast surgeries without ADM |
| Fakim et al., 2019 (20) | Artia  (LifeCell, NJ) | Animal tissue  (porcine) | Breast | 51  patients | Usage of Artia™ was correlated with low and satisfactory early complication rates. |
| Catellani et al., 2018 (21) | Braxon | Animal tissue  (porcine) | Breast | 84  patients | Pre-pectoral muscle-sparing ADM-wrapped implants resulted in lesser pain intensities and notable upper limb functional benefits in comparison with sub-muscular implant placements. |
| Powell et al., 2018 (22) | STRATTICE  Reconstructive Tissue Matrix (RTM) | Animal  Tissue  (porcine) | Breast | 84  patients | High levels of satisfaction and low levels of adverse effects including infection, seroma, and hematoma but high levels of skin flap necrosis |
| Lohmander et al., 2021 (23) | STRATTICE   pliable (Acelity, San Antonio, TX) | Animal  Tissue  (porcine) | Breast | 135  patients | Immediate use of ADM did not associate with fewer reoperations in comparison to implant-based breast reconstruction without ADM, and also satisfaction with aesthetic outcomes was equal in both groups. |
| Danino  et al., 201as9 (24) | AlloDerm  RTM | Human | Breast | 6 patients | The presence of bacterial biofilms on ADMs in all patients suffering from red breast syndrome was observed. |
| Levy et al., 2020 (25) | AlloMax | Human | Breast | 174  patients | Overall infection rates and time to drain removal were notably higher in the ADM group in comparison with the P4HB group, but rates of seroma were equal in both groups. |
| Brewer et al., 2010 (26) | AlloDerm | Human | Abdomen | 104 patients | They showed that ADM is associated with a lower recurrence rate compared to synthetic mesh. |
| Garvey et al., 2016 (27) | AlloDerm and SurgiMend | Human and bovine | Abdomen | 512 patients | Long-term results of abdominal wall reconstruction are improved by using ADM. |
| Butler et al., 2004 (28) | AlloDerm | Human | Abdomen | 19 Hartley guinea pigs | In abdominal hernia repair, visceral adhesion to the repair site is seen much more in the polypropylene method than when AlloDerm is used. |
| Han et al., 2010 (29) | Ruinuo | Human | Pelvis | 12 patients | ADM can be an appropriate choice for the reconstruction of large pelvic defects in the patients after cylindrical abdominoperineal resection. |
| Musters et al., 2016 (30) | Transperineal STRATTICE , Transperineal Permacol | Porcine | Pelvis | All consecutive patients who underwent a perineal hernia repair between March 2010 and April 2014 at the Academic Medical Center, Amsterdam | Using biological mesh repair of a perineal hernia after APR can lead to a high recurrence rate. |
| Coon et al., 2016 (31) | Alloderm, AlloMax, SurgiMend, Veritas, or STRATTICE | Human | Posterior trunk | 260 patients | an increased risk of infection and seroma with the use of biological tissue matrix in posterior trunk reconstruction is expected. Be aware of the possible risks and benefits of using this product. |
| Martell et al., 2009 (32) | AlloDerm^®^, | Human | Extremity (fascial of the lower limb) | 1 patient | The use of ADM in the fascial reconstruction of the lower limb had favorable results. |
| Cole et al., 2018 (33) | ArthroFlex^®^ |  | Extremity (Achilles tendon) | 9 patients | Tendon repair is successful with ADM due to the robustness of the minimally changed scaffold that gives additional repair stability, an ideal host for native cells, and a vessel ready to be replaced by native tissue. |
| Melandri et al., 2020 (34) | MODA (Matrice Omologa Dermica Acellulata) | Human | Extremity (extremity wounds with exposed tendons) | 1 patient | Tendon repair is successful with the use of an acellular human dermal membrane due to the robustness of the minimally changed scaffold that gives additional repair stability, an ideal host for native cells, and a vessel ready to be replaced by native tissue. |
| Lee et al., 2022 (35) | Insuregraf |  | Extremity (flexor tendon injury in hand Zones III, IV, or V) | 13 patients | The use of ADM in the repair of the flexor tendons of the III, IV, and V sections of the hand showed favorable results, including the prevention of peritendinous adhesions and the improvement of postoperative function. |
| Scalise et al., 2017 (36) | Integra^®^ | Animal  Tissue  (bovine & shark) | Extremity (plantar region) | 2 patients | Results of heel reconstruction using ADM, including optimal recovery of gait function and social participation. According to gait analysis, measured gait and posture are essentially normal. Nevertheless, the study of the pressure distribution reveals a small imbalance. |
| Fiedler et al., 2017 (37) | Singlelayer bovine acellular dermal matrix grafts (Thin; Integra Life Sciences, Inc., Plainsboro, NJ). | Animal  Tissue  (bovine) | Extremity  (nail bed) | 2 patients | Monolayer bovine acellular dermal matrix was used to reconstruct the sterile nail bed, but this kind of acellular dermal matrix grafting was not recommended for germinal matrix injuries to the nail bed. |
| Liu et al., 2020 (38) | Acellular dermal matrix (PELNACfi, Gunze Corp., Osaka, Japan) |  | Extremity  (nail bed) | 4 patients | Acellular dermal matrix and subsequent skin graft can be utilized to repair nail bed injuries and loss of germinal matrix. However, this approach may not be suitable for patients with complex crush trauma. |
| Askari et al., 2011 (39) | AlloDerm | Human | Extremity  (hand & wrist) | 9 patients | ADM can be utilized effectively in extremity burn repairs by minimizing wound contractions, lowering the risk of recurrence, and improving function. |
| Shang et al., 2021 (40) | Allogeneic |  | Extremity (hand) | 32 patients | ADM can be utilized effectively in extremity burn repairs by minimizing wound contractions, lowering the risk of recurrence, and improving function. |
| Cazzell et al., 2019 (41) | DermACELL |  | Lower extremities  (DFUs) | 61 patients | ADM has been deemed successful in closing DFUs. |
| Demircan et al., 2015 (42) | Collagen elastin matrix | Animal tissue (bovine) | Burn | 15 children | Graft quality was close to normal skin in terms of vascularity, elasticity, pliability, texture, and color.  Fast recovery with satisfactory aesthetic and functional results. |
| Heimbach et al., 2003 (43) | Integra™ | Animal tissue (bovine) | Burn | 216 patients | Integra helps in the treatment of burn wounds by reducing the possibility of invasive infection. |
| Okuno et al., 2018 (44) | PELNAC® | Human | Burn | 36 patients | It is useful for blocking invasive infections and the development of yeast in burn patients, based on the clinical results obtained. |
| Guo Z-Q, et al., 2016 (45) | Porcine ADM | Animal tissue (porcine) | Burn | 60 adult patients | Reduction of hospitalization time.  Wound healing.  Improving aesthetic and functional results. |
| Moiemen NS et al., 2010 (46) | Integra™ | Animal tissue (bovine) | Burn | 8 patients | The formation of hematoma and seroma, as well as shear forces, were greatly reduced with the use of TNP. |
| Bloemen MCT et al., 2010 (47) | Matriderm® | Animal tissue (bovine) | Burn | 46 patients | The surface of the scars is much smoother, which is very important in terms of aesthetics.  The autograft had given elasticity to the treated scars to a great extent. |

1. Helling ER, Dev VR, Garza J, Barone C, Nelluri P, Wang PT. Low fistula rate in palatal clefts closed with the Furlow technique using decellularized dermis. Plast Reconstr Surg. 2006;117(7):2361-5.

2. Mirzai S, Lee AH, Chi JJ. Nasal Septal Perforation Repair with an Inferior Turbinate Flap and Acellular Dermal Matrix. Surg J (N Y). 2021;7(1):e26-e9.

3. Bing Z, Feng L, Wu CS, Du JT, Liu YF, Liu SX. Acellular dermal matrix contributes to epithelialization in patients with chronic sinusitis. J Biomater Appl. 2019;33(8):1053-9.

4. Conrad DJ, Zhang H, Côté DWJ. Acellular Human Dermal Allograft as a Graft for Nasal Septal Perforation Reconstruction. Plast Reconstr Surg. 2018;141(6):1517-24.

5. Zhong B, Song NY, Deng D, Li LK, Du JT, Liu F, et al. Intraoperative Repair of Cerebrospinal Fluid Rhinorrhea in Skull Base Tumor Resection: A Retrospective Study of Acellular Dermal Matrix Versus Turbinate Flap. World Neurosurg. 2020;133:e275-e80.

6. Youngerman BE, Kosty JA, Gerges MM, Tabaee A, Kacker A, Anand VK, et al. Acellular dermal matrix as an alternative to autologous fascia lata for skull base repair following extended endoscopic endonasal approaches. Acta Neurochir (Wien). 2020;162(4):863-73.

7. Lee JM, Seo YJ, Shim DB, Lee HJ, Kim SH. Surgical outcomes of tympanoplasty using a sterile acellular dermal allograft: a prospective randomised controlled study. Acta Otorhinolaryngol Ital. 2018;38(6):554-62.

8. Park SC, Nam JS, Lee KI, Lee YW, Park JJ, Ha JG, et al. Effectiveness of cross-linked human acellular dermal matrix in primary and revision augmentation rhinoplasty. J Plast Reconstr Aesthet Surg. 2022;75(4):1447-54.

9. Yang CE, Kim SJ, Kim JH, Lee JH, Roh TS, Lee WJ. Usefulness of Cross-Linked Human Acellular Dermal Matrix as an Implant for Dorsal Augmentation in Rhinoplasty. Aesthetic Plast Surg. 2018;42(1):288-94.

10. Heo CY, Kang B, Jeong JH, Kim K, Myung Y. Acellular dermal matrix and bone cement sandwich technique for chest wall reconstruction. Arch Plast Surg. 2022;49(1):25-8.

11. Giordano S, Garvey PB, Clemens MW, Baumann DP, Selber JC, Rice DC, et al. Synthetic Mesh Versus Acellular Dermal Matrix for Oncologic Chest Wall Reconstruction: A Comparative Analysis. Ann Surg Oncol. 2020;27(8):3009-17.

12. Hansson E, Edvinsson AC, Elander A, Kölby L, Hallberg H. First-year complications after immediate breast reconstruction with a biological and a synthetic mesh in the same patient: A randomized controlled study. J Surg Oncol. 2021;123(1):80-8.

13. Dikmans RE, Negenborn VL, Bouman MB, Winters HA, Twisk JW, Ruhé PQ, et al. Two-stage implant-based breast reconstruction compared with immediate one-stage implant-based breast reconstruction augmented with an acellular dermal matrix: an open-label, phase 4, multicentre, randomised, controlled trial. Lancet Oncol. 2017;18(2):251-8.

14. Tierney BP, De La Garza M, Jennings GR, Weinfeld AB. Clinical Outcomes of Acellular Dermal Matrix (SimpliDerm and AlloDerm Ready-to-Use) in Immediate Breast Reconstruction. Cureus. 2022;14(2):e22371.

15. Negenborn VL, Smit JM, Dikmans REG, Winters HAH, Twisk JWR, Ruhé PQ, et al. Short-term cost-effectiveness of one-stage implant-based breast reconstruction with an acellular dermal matrix versus two-stage expander-implant reconstruction from a multicentre randomized clinical trial. Br J Surg. 2019;106(5):586-95.

16. Jones G, Antony AK. Single stage, direct to implant pre-pectoral breast reconstruction. Gland Surgery. 2019;8(1):53-60.

17. Brunbjerg ME, Jensen TB, Overgaard J, Christiansen P, Damsgaard TE. Comparison of one-stage direct-to-implant with acellular dermal matrix and two-stage immediate implant-based breast reconstruction-a cohort study. Gland Surg. 2021;10(1):207-18.

18. Khan A, Tasoulis MK, Teoh V, Tanska A, Edmonds R, Gui G. Pre-pectoral one-stage breast reconstruction with anterior biological acellular dermal matrix coverage. Gland Surg. 2021;10(3):1002-9.

19. Lohmander F, Lagergren J, Roy PG, Johansson H, Brandberg Y, Eriksen C, et al. Implant Based Breast Reconstruction With Acellular Dermal Matrix: Safety Data From an Open-label, Multicenter, Randomized, Controlled Trial in the Setting of Breast Cancer Treatment. Ann Surg. 2019;269(5):836-41.

20. Fakim B, Highton L, Gandhi A, Johnson R, Murphy J. Implant-based breast reconstruction with Artia (TM) tissue matrix. Journal of Plastic Reconstructive and Aesthetic Surgery. 2019;72(9):1548-54.

21. Cattelani L, Polotto S, Arcuri MF, Pedrazzi G, Linguadoca C, Bonati E. One-Step Prepectoral Breast Reconstruction With Dermal Matrix-Covered Implant Compared to Submuscular Implantation: Functional and Cost Evaluation. Clin Breast Cancer. 2018;18(4):e703-e11.

22. Powell-Brett S, Goh S. Clinical and patient reported outcomes in breast reconstruction using acellular dermal matrix. JPRAS Open. 2018;17:31-8.

23. Lohmander F, Lagergren J, Johansson H, Roy PG, Brandberg Y, Frisell J. Effect of immediate implant-based breast reconstruction after mastectomy with and without acellular dermal matrix among women with breast cancer: A randomized clinical trial. JAMA Network Open. 2021;4(10).

24. Danino MA, El Khatib AM, Doucet O, Dao L, Efanov JI, Bou-Merhi JS, et al. Preliminary Results Supporting the Bacterial Hypothesis in Red Breast Syndrome following Postmastectomy Acellular Dermal Matrix- and Implant-Based Reconstructions. Plast Reconstr Surg. 2019;144(6):988e-92e.

25. Levy AS, Bernstein JL, Xia JJ, Otterburn DM. Poly-4-Hydroxybutyric Acid Mesh Compares Favorably With Acellular Dermal Matrix in Tissue Expander-Based Breast Reconstruction. Ann Plast Surg. 2020;85(S1 Suppl 1):S2-s7.

26. Brewer MB, Rada EM, Milburn ML, Goldberg NH, Singh DP, Cooper M, et al. Human acellular dermal matrix for ventral hernia repair reduces morbidity in transplant patients. Hernia. 2011;15(2):141-5.

27. Garvey PB, Giordano SA, Baumann DP, Liu J, Butler CE. Long-Term Outcomes after Abdominal Wall Reconstruction with Acellular Dermal Matrix. J Am Coll Surg. 2017;224(3):341-50.

28. Butler CE, Prieto VG. Reduction of adhesions with composite AlloDerm/polypropylene mesh implants for abdominal wall reconstruction. Plast Reconstr Surg. 2004;114(2):464-73.

29. Han JG, Wang ZJ, Gao ZG, Xu HM, Yang ZH, Jin ML. Pelvic Floor Reconstruction Using Human Acellular Dermal Matrix After Cylindrical Abdominoperineal Resection. Diseases of the Colon & Rectum. 2010;53(2):219-23.

30. Musters GD, Lapid O, Stoker J, Musters BF, Bemelman WA, Tanis PJ. Is there a place for a biological mesh in perineal hernia repair? Hernia. 2016;20(5):747-54.

31. Coon D, Calotta NA, Broyles JM, Sacks JM. Use of Biological Tissue Matrix in Postneurosurgical Posterior Trunk Reconstruction Is Associated with Higher Wound Complication Rates. Plast Reconstr Surg. 2016;138(1):104e-10e.

32. Martell MEG, Kakar BY, Muschett DM, Armstrong MB. Lower Extremity Fascial Reconstruction Using an Acellular Dermal Matrix Graft. Wounds. 2009;21(5):124-6.

33. Cole W, Samsell B, Moore MA. Achilles tendon augmented repair using human acellular dermal matrix: a case series. The Journal of Foot and Ankle Surgery. 2018;57(6):1225-9.

34. Melandri D, Marongiu F, Carboni A, Rubino C, Razzano S, Purpura V, et al. A new human-derived acellular dermal matrix for 1-stage coverage of exposed tendons in the foot. The International Journal of Lower Extremity Wounds. 2020;19(1):78-85.

35. Lee YJ, Ryoo HJ, Shim H-S. Prevention of postoperative adhesions after flexor tendon repair with acellular dermal matrix in Zones III, IV, and V of the hand: A randomized controlled (CONSORT-compliant) trial. Medicine. 2022;101(3).

36. Scalise A, Torresetti M, Verdini F, Capecci M, Andrenelli E, Mengarelli A, et al. Acellular dermal matrix and heel reconstruction: a new prospective. Journal of Applied Biomaterials & Functional Materials. 2017;15(4):e376-e81.

37. Fiedler DK, Barrett JE, Lourie GM. Nail bed reconstruction using single-layer bovine acellular dermal matrix. The Journal of Hand Surgery. 2017;42(1):e67-e74.

38. Liu T-H, Hsieh M-C, Chou P-R, Huang S-H. Reconstruction for defects of total nail bed and germinal matrix loss with acellular dermal matrix coverage and subsequently skin graft. Medicina. 2020;56(1):17.

39. Askari M, Cohen MJ, Grossman PH, Kulber DA. The use of acellular dermal matrix in release of burn contracture scars in the hand. Plastic and reconstructive surgery. 2011;127(4):1593-9.

40. Shang F, Hou Q. Effects of allogenic acellular dermal matrix combined with autologous razor‐thin graft on hand appearance and function of patients with extensive burn combined with deep hand burn. International Wound Journal. 2021;18(3):279-86.

41. Cazzell S, Moyer PM, Samsell B, Dorsch K, McLean J, Moore MA. A prospective, multicenter, single-arm clinical trial for treatment of complex diabetic foot ulcers with deep exposure using acellular dermal matrix. Advances in Skin & Wound Care. 2019;32(9):409.

42. Demircan M, Cicek T, Yetis MI. Preliminary results in single-step wound closure procedure of full-thickness facial burns in children by using the collagen-elastin matrix and review of pediatric facial burns. Burns. 2015;41(6):1268-74.

43. Heimbach DM, Warden GD, Luterman A, Jordan MH, Ozobia N, Ryan CM, et al. Multicenter postapproval clinical trial of Integra dermal regeneration template for burn treatment. J Burn Care Rehabil. 2003;24(1):42-8.

44. Okuno E, Jarros IC, Bonfim-Mendonça PS, Vicente de Rezende G, Negri M, Svidzinski TE. Candida parapsilosis isolates from burn wounds can penetrate an acellular dermal matrix. Microb Pathog. 2018;118:330-5.

45. Guo ZQ, Qiu L, Gao Y, Li JH, Zhang XH, Yang XL, et al. Use of porcine acellular dermal matrix following early dermabrasion reduces length of stay in extensive deep dermal burns. Burns. 2016;42(3):598-604.

46. Moiemen NS, Yarrow J, Kamel D, Kearns D, Mendonca D. Topical negative pressure therapy: does it accelerate neovascularisation within the dermal regeneration template, Integra? A prospective histological in vivo study. Burns. 2010;36(6):764-8.

47. Bloemen MCT, van Leeuwen MCE, van Vucht NE, van Zuijlen PPM, Middelkoop E. Dermal substitution in acute burns and reconstructive surgery: a 12-year follow-up. Plast Reconstr Surg. 2010;125(5):1450-9.
